# Foreign Medical Literature

**Published:** 1800-06

**Authors:** 


### FOREIGN MEDICAL LITERATURE.

### AMERICAN.

We have received the New York Medical Repoſitory up to January laſt, from which we extract the following Articles:

*A Dissertation on the bilious malignant Fever which prevailed in the Country adjacent to Dartmouth College, New Hampſhire, in the Summer of* 1799; by Lyman Spalding, M. B.

"In the cure of this fever, ſome hurtful and *many* uſeleſs applications were made. It was not in the leaſt effected by thoſe applications which are moſt extolled in the fevers of our climate. Moſt of the practitioners had never ſeen it before, and thoſe who had, from motives of prudence, were unwilling to acknowledge its identity with that of Philadelphia.

"On the firſt attack, an emetic, adminiſtered in ſmall doſes, to operate cathartically, relieved the ſymptoms. The effervеſcing mixture given in the hot fit gave univerſal relief. New beer and aceſcent potations were highly grateful.

"Calomel, joined with other cathartics, was much uſed. Bliſtering, and mercurial unguent rubbed over the whole body, were uſeful.

"Cold air was highly grateful, and eagerly ſought for. To eſtabliſh a current of air through the room was of the firſt conſequence: all the windows and doors were kept open, and the air put in motion by fanning. The rooms were conſtantly moiſtened with vinegar, or vinegar and water. Cleanlineſs was of the firſt conſequence: the patients were frequently waſhed all over with vinegar and water, accompanied with friction: a ſolution of muriate of ſoda was ſometimes made uſe of. Putrid ſtools were inſtantly removed, and the ſtench corrected. All unneceſſary apparel and furniture were removed.

"But the moſt dependence was placed upon the *cold bath,* when the hot fit was on. This infallibly gave inſtant and aſtoniſhing relief, rendering the paroxyſms ſhorter and milder. It was applied either generally or locally, as the urgency of the caſe required: this was determined by the ſurface that appeared unuſually hot. When the heat was partial or local, a correſponding bath was uſed.

"The

"The cold bath was generally applied, by laying the patient naked upon a thick blanket, then ſprinkling him and the blanket with the coldeſt water: the wetted blanket was wrapped around him, and ſuffered to remain till it became warm; when it was thrown off, and ſprinkled a ſecond time; thus reducing the heat of the body to the ſtandard of health. The cold bath ſerved only till the criſis of the fever; afterwards it was as diſtreſſing and painful as heretofore it was invigorating and pleaſant. Many patients have been injured by a continuation of the bath after the criſis. Waſhing the hands and face in cold water was grateful.

"Bleeding did not produce that good effect which we had been taught to expect. I am ſupported in the opinion by phyſicians grown grey in the uſe of the lancet, that it was, in every inſtance, in all ſtages of the fever, *evidently injurious.* Neither was it found neceſſary to procure any ſudden evacuations; for *here* the diſeaſe ſcarcely terminated in the ſame number of weeks as hours at Philadelphia. Warm bathing was hurtful, except to the cold extremities.

"Nitre, and other refrigerants and febrifuges had no laſting effect; opiates had not till after the criſis, neither had bark: this was now uſed with a liberal hand. A watery diarrhœa was the moſt troubleſome and pertinacious ſymptom.

"The *chief indications* of cure, as delivered by my worthy friend Dr. Smith, in his courſe of lectures on the theory and practice of phyſic, are, to regulate the heat of the body according to the ſtandard of health, and ſupply the ſyſtem with ſuch ſubſtances as readily yield carbone."

---

*A Case of Tetanus cured by Wine*; by Dr. D. Hossack.

"On Tueſday, March 13, 1798, about one o'clock P. M. I was called to viſit a mulatto ſervant woman of John Harrington, Eſq. of this city. I was informed that about an hour before, while engaged in waſhing clothes, ſhe had pricked herſelf with a pin in the wriſt of her right-arm. The part at which the pin entered was upon the inſide of the wriſt, immediately over the connection of the radius with the carpus.

"The pin was inſtantly removed, and, finding no inconvenience from the accident, ſhe returned to her employment. In a ſhort time ſhe felt a great degree of ſoreneſs in the part which had been injured, with pain ſhooting occaſionally to the arm, ſhoulder and neck. Theſe ſymptoms, in a few minutes, were ſucceeded by ſtiffneſs about the throat, difficulty of ſwallowing, ſome interruption of her ſpeech, and, at length, a locked ſtate of the jaws, attended with a ſpaſmodic contraction of the muſcles at the back part of the neck, and occaſional ſubſultus tendinum, with ſome coldneſs of her extremities. In this ſituation I found her.

"She was naturally of a delicate and irritable habit of body, and had been much ſubject to hyſterical complaints and fits of fainting, which were ſometimes induced by the moſt trifling cauſes. Her irritability of habit was alſo at this time probably increaſed, having but three months before borne a child, which ſhe was then ſuckling.

"Although I have been long ſince convinced of the inſufficiency of opium in the cure of this diſeaſe, in the hurry of the moment I gave her about ſixty drops of laudanum, in a ſmall quantity of wine. Her jaws being cloſely locked, it was with great difficulty adminiſtered. In a few minutes after ſwallowing the laudanum ſhe ſickened at the ſtomach, and vomited violently, complaining at the ſame time of great pain and diſtreſs at the pit of her ſtomach. The anodyne draught was entirely rejected; but, upon a moment's reflection, I did not regret this circumſtance, as the diſeaſe aſſumed a very decided character, and I had made up my mind to rely upon the effects of wine alone, without the aſſiſtance of any other remedy: accordingly, about two o'clock, I directed a large wine-glaſs full of *Madeira wine* (the glaſs containing about two ounces), to be given punctually every hour, and a cup of ſago, or panado, with wine, to be given, from time to time, as her nouriſhment. At this time another phyſician, who had alſo been called upon at the time of the accident, arrived. I related to him what had been done, and the mode of treatment which I directed for the patient. This gentleman having had frequent opportunities of ſeeing this diſeaſe, and having frequently witneſſed the failure of the ordinary mode of treatment, he at once, with great candour, acceded to the plan propoſed, and, in addition to the uſe of wine, propoſed the application of cauſtic to the part which had been wounded. Accordingly, the wound was freely pencilled with the lunar cauſtic, and afterwards covered with a poultice of bread and milk, with the view to obtain ſuppuration as ſoon as poſſible.

"The wine was adminiſtered with great fidelity by the mother of the patient, until about five o'clock the next morning. She had ſome ſlight convulſions in the courſe of the afternoon, but they were more of an hyſterical ſort, induced by her great anxiety of mind, than to be aſcribed to the diſeaſe itſelf. Generally ſpeaking, there had been a very manifeſt abatement in all her ſymptoms, and ſhe had paſſed a more comfortable night than could have been expected. At five o'clock on Wedneſday morning, her miſtreſs, alarmed at the quantity of wine ſhe had taken, deſiſted from its further uſe. From this time, appearances became more unfavourable, and at eight o'clock her jaws, which had been relaxed during the plentiful uſe of wine, again became ſtiff and cloſed. We ſaw her at nine, and immediately gave her about half a pint of wine, and ordered it to be adminiſtered as before. At one her ſymptoms were greatly changed; we found her ſitting up in bed, eating ſmall portions of roaſted oyſters, which ſhe had called for. At this time her jaws were almoſt in their natural ſtate. She had taken her wine punctually as directed, but experienced no inconvenience from it whatever, although in health ſhe had not been accuſtomed to its uſe. Her pulſes were ſtill ſmall and feeble, without any excitement from the uſe of wine. The heat of body remained at its natural ſtandard, but not at all increaſed. The pain in her head was abated, but but without any appearance of suppuration. Finding this mode of treatment to agree so well with her, we directed it to be continued. We saw her again in the evening: her symptoms still continued favourable, without the smallest febrile action from the use of wine. Having had no discharge from her bowels since her illness, an injection was administered; which remedy was afterwards employed from time to time in the course of her disease, whenever the state of her bowels required it. The wine was continued through the night: she slept, altogether, about three hours in the course of the night; and took freely of her panado.

"Thursday morning at nine o'clock, her complaints appeared to be, in a great measure, subdued; insomuch that we did not think it necessary to visit her again until late in the evening, and directed the wine to be given at longer intervals, and the quantity to be lessened.

"She remained in a very comfortable condition until the afternoon—the *pain in her hand returned* with violence, extending to her arm and neck as before—her jaws were again closed—the rigidity of the muscles at the back of her neck returned—her mind became greatly agitated—she again complained of distress at the pit of her stomach—she fainted, and had several slight convulsions. Being called at that time, I gave her, with some difficulty, about half a pint of wine, and ordered a warm poultice to be immediately boiled. When prepared, I poured upon the surface of it, half an ounce of laudanum, and applied it to the wound. Her symptoms were in a short time allayed: I left her, directing the wine to be continued as before, a large wine-glass full every hour.

"We saw her again at nine in the evening. She remained tranquil—her jaws were less firmly closed, but the pain in her hand was not altogether removed. Although she had taken the wine punctually as directed, it had not produced the least apparent excitement. Having had no discharge from her bowels for the last twenty-four hours, an injection was administered. The anodyne poultice was renewed; and, in addition to this application, we directed her arm to be bathed with laudanum occasionally through the night.

"*Friday morning* we found she had passed a more comfortable night than the last; had taken her wine every hour; her jaws were perfectly relaxed; the pain in her hand had greatly abated, and she was enabled to extend her fingers at pleasure, which she could not do before. Her pulses and skin were natural; her appetite unimpaired; her mind composed, without any inconvenience from the wine. We directed her remedies to be all continued as before, fearing lest any alteration might subject her to a return of her complaints.

"In the evening we observed the wine had exhilarated her spirits; she became very talkative; her pulses became full, and free from all tension; her skin was somewhat heated, and all complaints removed except the wound at the wrist, which exhibited a healthy appearance, and was entirely free from pain, but without any sign of suppuration.

"We

"We directed the wine to be adminiftered through the night, but in fmaller quantities and at longer intervals, unlefs her complaints fhould return and demand a continuance of it as before.

"Saturday morning we were informed fhe had flept the greater part of the night, and had taken but a fmall quantity of wine; her fymptoms being, in all refpects, favourable, the wine was difcontinued, except a fmall quantity mixed with nourifhment. A dreffing, of fimple ointment, was applied to the wound. From that time fhe remained free from any return of her complaints, and has fince been in perfect health.

"Upon calculating the quantity of wine which fhe had taken, it amounted to three gallons."

[Dr. Noehden informs us, that Dr. Stütz, of Swabia, has fucceeded in curing this dreadful difeafe, by the ufe of a hot bath, impregnated with kali and a few ounces of quick lime; and giving internally ten grains of kali, and one of opium, every two hours. We merely mention this circumftance at prefent, as Dr. Stütz gives us hopes that he will treat this fubject more explicitly in his Mifcellaneous Medical Obfervations, which will foon be publifhed.]

---

*Two Cases of the human Constitution being affected by the Contagion of Small-Pox and Measles at the same Time*; by P. Tracy, M. D.

"In the fpring of the year 1797, being then engaged in inoculating for the fmall-pox, two cafes fell under my care, which exhibited unequivocal evidence of the poffibility of two diftinct difeafes arifing at the fame period in the human frame, and each purfuing its ordinary courfe as when feparately exifting, attended with all their ufual characteriftic marks; and though I am fenfible that the weight of medical opinion may militate againft my experience on this queftion, ftill the conclufive evidence that the facts afford, has removed every doubt that previoufly exifted in my mind on contemplating the fubject, and leads me to cheerfully fubmit the cafes to the candour of the faculty.*

"Cafe 1. W. T. a young man, applied for admiffion into my hofpital for inoculation, and mentioned, at the fame time, his having been expofed, a day or two previoufly, to take the meafles, which excited fome anxiety in his mind, refpecting the fafety of receiving the fmall-pox, under the liability of being affected with the meafles at the fame period. Thinking this a good opportunity to determine, whether two fpecific contagions could operate at the fame time on the human frame, and concluding no great danger would attend the experiment, I received him into the hofpital, and inferted the variolous matter, from a well fuppurated puftule, in the ufual manner. The local inflammation, at the part inoculated, came forward at the common period, with as much activity as occurred on the other patients, at the fame time inoculated; which was

---

* See Journal, Vol. II. p. 27.

was followed by the precurfory eruptive fever on the eighth day from inoculation. The fymptoms were mild, and continued to the tenth day, at which period a number of diftinct puftules were vifible around the place inoculated, and many were difcoverable on the face and neck, juft emerging from the fkin; the eruptive fymptoms continued ftill in a moderate degree. The mild form which the variolous difeafe at this time affumed, greatly relieved my patient from his anxiety, arifing from the fearful apprehenfion of being jointly attacked with two fo formidable difeafes as the fmall pox and meafles, at the fame period. I left him in this ftate of tranquillity; but in about four hours after I was called to vifit him, and found him labouring under fevere pain in the head and loins, attended with rigors, pyrexia, &c. As he had not expofed himfelf to take cold, or been guilty of any marked imprudence, I immediately fufpected him affected with the premonitory fymptoms of meafles; and, in conformity with this belief, adopted phlebotomy, with the antiphlogiftic method generally. On the next day, the meafley efflorefcence made its appearance on the furface, attended with cough, coryza, and all the other ufual marks of this difeafe, which progreffed in the common manner to a favourable iffue. During this period, from the firft acceffion of the meafley fymptoms, the local inflammation at the part inoculated continued bright, and the previous puftules not only remained vifible, and progreffing towards maturation, but a number of additional puftules actually appeared on the lower extremities, eafily diftinguifhable by their hardnefs and prominency, and which maturated in the ufual manner as when feparately exifting, except that the fuppuratory procefs feemed lefs rapid than in many other cafes: thus, hand in hand, thefe two diforders proceeded to a favourable termination, which freed my patient from his great folicitude, and impreffed me with the belief, that different fenfitive principles of the animal frame may be morbidly excited, from different caufes, at the fame period, each equally productive of its peculiar form of difeafe.

"Cafe 2. J. S. after being expofed to the fmall-pox, in the natural way, was feized, at the ufual period, with the fymptoms of the diforder, which were followed by a puftular eruption on the furface; and, on the next day from their firft appearance, was attacked with fymptoms of a fimilar afpect to thofe which fupervened in the former cafe, though in a more aggravated degree, which I treated as in the former inftance, and which were followed by an univerfal meafley eruption on the third day, with the ufual concomitants. During this period, the previous puftular eruption, which was copious, remained bright and prominent, and new puftules continued to appear on the lower extremities, all of which proceeded to maturation in the ufual manner; while the meafles purfued their ordinary courfe, neither difeafe feeming to retard the other in its progrefs, but, like two friendly fojourners in feparate apartments of one tenement, feemed mutually difpofed to purfue their different careers, without officious interference or moleftation to each other. This cafe, like the other, terminated happily. No room was left to

 doubt doubt the identity of the meafles in either cafe, as neither of the fubjects had previoufly been affected with that diforder: both had been expofed to take it: and a confiderable number of my patients at the hofpital, without having been fenfibly expofed to the difeafe in any other way but from thefe patients, were affected at the ufual time with this diforder.

"Feeling myfelf greatly incompetent to explain the variant fenfitive principles in the human machine, which are fubject to be conjunctly excited from different caufes, each productive at the fame time of the phenomena, peculiarly marking diftinct diforders, I fhall avoid the attempt; but this belief is ftrongly impreffed on my mind, that two or more fpecifically different diforders may arife at the fame time in the human frame, and purfue their natural courfes, notwithftanding the zeal with which the Brunonian advocate attempt to fupport the untenable theory of the unity and indivifibility of their principle of excitability pervading the fyftem at large, and being fufceptible of only an individual morbid excitement at a time."

*Case of the deleterious Effects of Opium remedied by the Excitement of Pain.* By V. Seaman, M. D.

"Having fo frequently obferved the great quantity of opium that a perfon under acute pain will take, without having any foporific effects induced by it, I have long been of the mind, that pain might be ufefully excited to remove the deadly influence of a large dofe that may have been previoufly taken. This idea I intimated in my Inaugural Differtation, publifhed in 1792.

"Yefterday (July 2, 1799) I had an opportunity of putting my principles to the teft of experiment, being called to fee the wife of —— Head, in Water-ftreet, who had, about two hours before, taken an ounce of laudanum, and then lay in a deadly ftupor, from which all the efforts of her friends were infufficient to awaken her. Attempts had been made to get fome vinegar into her ftomach, but, I believe, with little effect; nor did I fucceed much better in endeavouring to give her a dofe of white vitriol. I then procured a fmall fwitch, and applied it pretty freely to her arms and fhoulders, which were defended only by a thin linen covering. I alfo applied fome ftrokes to her legs. In the courfe of a very fhort time, indeed almoft immediately upon the application of this remedy, fhe roufed up, and begged me to defift. She continued for a time much confufed, with involuntary fits of laughter. Two fcruples of white vitriol were then adminiftered, followed in about fifteen minutes by half a drachm of ipecacuana; notwithftanding which, and alfo having her throat tickled with an oiled feather, it was near an hour before fhe could be made to puke; however, finally, fhe puked, and by the affiftance of frequent draughts of warm water, her ftomach was pretty thoroughly evacuated.

"By the affiftance of her friends fhe was kept awake, or, at leaft, flept but little at a time during the night, and this morning appears entirely recovered."

GERMANY,

### GERMAN.

*Dr. Lentin's Obſervations on the Dolor Faciei.* (Extracted from Hufeland's Practical Journal, Vol. IX. No. 1. p. 56.)

THE *Dolor Faciei*, or, as the French call it, the *Tic Douloreux*, is a diſorder which has, in general, fruſtrated all attempts of the medical art; no medicine hardly has relieved the diſeaſe, and inſtances of a permanent cure have very rarely, if ever, occurred. This terrible complaint has a peculiarity whereby it materially differs from gout and rheumatiſm, viz. that the moſt vehement pain is brought on by touching, in the ſlighteſt way, the affected part at the period of its furious paroxyſm. Some patients were obliged to ſubdue their appetite for a time, being fearful of indulging it by movement of the lips, by chewing, &c. Others, to procure relief, uſed to rub their cheeks, where it generally has its ſeat, in ſuch a vehement manner, that they became quite callous. In one inſtance the diſeaſe ended in madneſs; in another, the patient got a little relief, after having ſuffered above eleven years; but great indurations of the glands of the inteſtines came on, of which he died; a third perſon, that was affected in this way, got a cancer in the mouth, and on the tongue. Dr. Lentin met, in the courſe of a twenty-ſeven years practice, with fourteen patients afflicted with this horrid malady; and he candidly confeſſes, that he never could boaſt of having performed a permanent cure. The remedy he found particularly efficacious, was the tinctura ſtrammonii (Datura Strammonium, L.) ℞. Seminum ſtrammonii unc. ij. vin. Hiſpanici unc. viij. ſpirit. vin. unc. j. digere per aliquot dies et filtra; doſis gutt. vj. Beſides this, ſulphureous baths, particularly that of Nendorf, in the dominions of the Landgrave of Heſſen Caſſel, proved very uſeful.

Dr. Lentin further obſerved ſome curious varieties of this evil; in one caſe the ſeat of it was in the foot, and a ſmall piece of paper falling upon it, would excite the pain for ſeveral hours. Another remarkable variety was obſerved by him in a lady, who otherwiſe enjoyed very good health: She felt a violent pain in the right ſide of her head, whenever ſhe undertook any buſineſs which required ſome attention, or a rapid tranſition from one idea to another. She could not, for ſome time, hear well with the right ear, but this defect was remedied by the application of a ſtrong magnet. She was much afflicted at the loſs of her huſband, whom ſhe tenderly loved, and, as long as the violence of her grief remained, ſhe was not at all troubled by the complaint; but her grief being diminiſhed, the pain gradually became more vehement and laſting. When her attention was engaged by a lively converſation ſhe found a ſhort relief, but afterwards the evil returned with renewed force, and with a violence, greater in proportion than the alleviation ſhe had experienced. No ſymptoms of ſpaſm could ever be traced, nor were antiſpaſmodic remedies of any avail; even the tinctura ſtrammonii was uſeleſs; and, in ſhort, every poſſible remedy was given in vain. She ſeemed, however, to be in a blooming ſtate of

 health.

health. In her youth ſhe had had ulcerated glands on both ſides of the neck, of which ſeveral ſcars were remaining along the jugular veins; and this might be conſidered as a ſufficient cauſe by obſtructing the eaſy reflux of the blood from the head, if the pain had not commenced long after the cicatrization of the ulcers.—The diſeaſe is not likely to be of a cancerous nature, at leaſt in its early periods, elſe the patients could not rub the parts immediately affected, ſo vehemently, without ill conſequences. The patients are much influenced by dry and wet weather.

---

*On the Uſe of the Oleum Hyoſcyami in Hæmoptyſis.* (Hufeland's Journal, Vol. IX. No. 2.)

Prof. Harles, of Erlangen, recommends the internal uſe of the oleum hyoſcyami as one of the beſt, ſureſt, and mildeſt remedies to ſtop an hæmoptyſis. He diſtinguiſhes two principal ſpecies of that diſeaſe; the firſt originates in an exceſſive irritability, irritation, and more or leſs ſpaſmodic action of the arteries of the lungs, which bring on an extravaſation of blood into the air cells, and a rupture of the ſmall blood veſſels follows. The ſecond conſiſts in a diminiſhed irritability and action of the veſſels, from their debility and relaxation, by which a rupture of their membranes, and an effuſion of blood, is occaſioned. It is in the firſt the oil of hyoſcyamus is indicated, as well as the extract of it, which has been much commended by other German phyſicians, though it has by no means ſo ſure and quick an effect as the oil of the ſame plant. In moſt caſes this has ſtopped hæmoptyſis after a few doſes have been taken; which either did not return at all, or was eaſily ſuppreſſed by the ſame remedy. Sometimes it has been neceſſary previouſly, to take a little blood from the patient. The mode of preparing the oil is to boil two ounces of freſh ſqueezed leaves of hyoſcyamus niger, L. in ounces of pure ſweet oil for a little time. One ounce of this to be mixed with two ounces of Caſtor oil, or oil of ſweet almonds, and four tea ſpoons full to be given as a doſe four times a day.

---

### PHARMACOLOGY.

*Pharmacologiae Univerſae, part I. quam in usum auditorum ſuorum concinnaverat* F. T. Valtelen, dum in vivis eſſet, M. D. Med. et Chimiae in Academ. quae Leidae eſt, Profeſſor ordinarius. Lugduni, Batavorum apud Van Thoir, 1797,8, pp. 400.

This work, which made its appearance after the author's death, deſerves, undoubtedly, to be ranked amongſt the better productions of the kind, as it in general anſwers the requiſites of a good practical Pharmalogy. The whole of the work is divided into four heads. 1. *Indicationum Doctrina;* 2. *Univerſa Materia Medica S. Pharmacologia*; 3. *Pharmacia Chemica;* 4. *Regulae ſecundum quas* formulae medicamentorum conſignari et ex arte praeſcribi debent. The preſent volume contains only the firſt diviſion and a part of the the fecond, 1. *Indicationum doctrina,* from p. 15 to 84. Here the fundamental therapeutic principles are propofed, particularly with refpect to the doctrine of indications; to which is added a divifion of the remedies into claffes, according to their therapeutic effects; and the cafes are exactly defcribed and determined, which either require or reject the ufe of each clafs of remedies. It is not neceffary to give a full account of the contents of this chapter, as it merely treats of therapeutic principles, which we muft fuppofe to be fufficiently known to our readers; and we only wifh the author had taken more notice here of the modern principles of medicine, the doctrine of the vis vitalis, &c. by which the medical art has of late undergone fo many improvements. 2. *Pharmacologia Univerfa.* With this divifion begins Pharmacology, properly fo called, wherein the fimples and compounds are enumerated according to their qualities and ufes, in a concife and eafy ftile, and in the following order:

I. Vegetabilia. 1. Farinofa, Mucilaginofa f. Gummofa; 2. Aquosa, Subdulcia; 3. Pinguia Oleofa Subdulcia; 4. Dulcia vifcofa; 5. Acida et Acido dulcia; 6. Alcalina Vegetabilium falia; 7. Media Vegetabilium falia; 8. Auftera, Adftringentia; 9. Amara et Amaricantia; 10. Fragantia, Aromatica, Balfamica, Refinofa; 11. Acria, Cauftica; 12. Acria et Amara, Emetica et Cathartica; 13. Acria virofa, Narcotica; 14. Vinum et Spiritus ardens.

II. Fossilia. 1. Terrae; 2. Salia Acalina; 3. Salia acida; 4. S. media; 5. Sterreftria; 6. Bitumina; 7. Metalla; 8. Semimetalla.

III. Animalia horumque partes. 1. Terreftria et Terreogelatinofa; 2. Glutinofa, Pinguia, Oleofa; 3. Amara; 4. Acria Cauftica; 5. Graveolentia Curationes per reliqua inftrumenta.

IV. Aqua. 1. Aqua fincera fluida; 2. A. vaporafa; 3. Nix et Glacies; 4. Aqua medicata mineralis.

V. Aer. 1. A. communis falutaris et noxius; 2. A. medicatus varius, a. Fumigatione, b. Odoribus; 3, Inflatio more Ægyptiorum; 4. Gas multiplex.

VI. Ignes. 1. Flamma; 2. Carbo; 3. Cauterium; 4. Electricitas.

VII. Mechanica Remedia. 1. Venæfectio; 2. Scarificatio; 3. Cucurbitulae cruentae; 4. Hirudines; 5. Clyfma; 6. Setacea et Fonticuli; 7. Flagellatio et Urticatio; 8. Frictio; 9. Ligatura; 10. Motus; 11. Inoculatio; 12. Magnetifmus. As the remedies contained in this firft volume comprehend only the firft eight claffes of the vegetable kingdom, we have ftill to expect a confiderable number of volumes.

Among the great number of books we poffefs on Materia Medica, the prefent certainly obtains a very honourable place. Though it affords nothing new, or reprefented in a new view, yet, the accurate determination of the cafes in which the different remedies are to be given, the continual quotations of the moft approved practitioners, render this book very commendable to the medical ftudent. The only thing to which we have to object is, the order which has been followed by the author, as it is by no means calculated to give a furvey of the different remedies according cording to their essential properties and original virtues, though this should be particularly attended to. For, by dividing the remedies according to the regna naturae, as has been adopted by the author, a separation of remedies, otherwise perfectly agreeing with each other, is unavoidable; and hence arises the disagreeable necessity to look for medicines, which are of the same kind, under different divisions, viz. vegetable and mineral alkalies, vegetable and animal bitters, &c.; and farther, no proper place can be given to remedies composed of substances belonging to different regna naturae. Besides this, it would not have been improper to prefix to each class of remedies, a general account of their effects and properties, as this certainly facilitates the attainment of the difficult study of Pharmacology to the beginner.

### BOTANY.

*Lichenographiae Suevicae Prodromus.* Auctor Erick Acharius, M. D. Medicus Provincialis Ostro-Gothiae, etc. Lincopiae, 1798, pp. 264, 8vo. with two coloured plates, price 7s.

The number of Lichens found in Sweden has been considerably increased since the publication of Linnæus's Flora Suevicae. Mr. Acharius made them, a long time since, a particular object of his botanical study, and his merits in this family of Cryptogamic plants, are already sufficiently known by his former publications. The present work is a new pledge of his knowledge in the lichens, and certainly deserves the attention of every botanist. It contains more than one might expect from a Prodromus, and is full of interesting remarks, and far from being merely a dry catalogue. The distribution of the lichens he has followed here, is according to the Truneus, and he divides them therefore, into three families: 1. *Crustacei;* 2. *Foliaces;* 3. *Caulescentes;* each of them is again divided into several *tribes*, of which there are twenty-eight altogether, and a proper name is given to every tribe. The author has, besides, carefully attended to collect all synonymous terms, and every where referred to the best figures. Those lichens which are not met with in Sweden, he has enumerated at the end of each tribe. The whole work comprehends 529 species, 101 of which are new, and not yet described. The Swedish lichens amount to 345; the rest are not found in that country. A useful register concludes the work. We have purposely not given a more accurate account of this work, as we suppose it will come to the hands of every botanist who interests himself about the extensive and difficult class of lichens.

### MEDICINE.

*Die Neuesten Entdeckungen, etc.* i. e. The latest Discoveries and Illustrations in Medicine, systematically arranged. By F. L. Augustin, M. D. Physician at Berlin, &c. vol. I. for 1798, 8vo. 564 pp. 1799. Berlin, Felish.

The plan of the author is to collect yearly all discoveries and improvements in the medical art, and to represent them concisely and and as faithfully as poſſible in a yearly publication. We muſt confeſs, that the author has ſhown a great deal of diligence and learning in the different branches of the medical art; and we are, moreover, convinced that this undertaking will prove very uſeful, particularly to thoſe that neither have time nor opportunity to read and ſtudy all the publications in medicine, which appear from year to year.

The work is divided into two principal parts. The firſt treats of medicine in general; of the methodology of medicine, or medical theory, according to *Roeſhlaub,* &c. The ſecond is divided again into two ſections, of which the firſt contains from p. 13 to 174, what belongs to the ſound ſtate of the body; the ſecond, to the end of the volume, what has been ſtated of the diſeaſed ſtate of the body. At the end of this volume, all the writings are enumerated, of which the author availed himſelf in the compoſition of this book, and to which he has referred by numbers: They amount to 522, including the journals, ſingle numbers of them, ſmall pamphlets, and diſſertations. The firſt ſection has three chapters; 1. *Of the doctrine of organiſation*; 2. *Of the doctrine of vis vitalis, or phyſiology properly called*; and, 3. *Of diatetic.* The neweſt chemical and anatomical diſcoveries are mentioned here, relating to organization, Roeſhlaub's, Reil's, Hufeland's, &c. Ideas on vis vitalis and phyſiology are propoſed, with a ſtatement of the progreſs of galvaniſm and magnetiſm, according to Stumboldt, Ritter, &c. Of the diſcoveries in diatetic, he mentions Mr. Plouquet's water-bed, which conſiſts of a frame of wood, acroſs which are firſt nailed a number of ſtraps, ſufficient to ſupport a kind of linen or flannel hammock, which is to be faſtened above them; on that the perſon lies; the whole is hung on poles in a convenient part of a river. The propoſals for the diet of different ſituations of life, are likewiſe related; viz. the diet of ſoldiers, according to *Blair* and *Gilbert*; of ſeamen, according to Stewart, Arthy, and Trotter; of the miners, according to Kortum; of actors, according to Stunnius: The diet for women contains every thing belonging to the obſtetric art. An Appendix is here added on Brown's diet, according to *Roeſhlaub.* The ſecond ſection comprehends, under the title of *Noſodick,* 1. *Pathology*; definitions of diſeaſe, diſtributions of diſorders, a critical review of the Brunonian principles, obſervations on organic diſeaſes, &c. 2 *Therapeutic,* which is treated in three diviſions, *Introduction, Materia Medica,* and *Special Therapeutic.* But we ſhall not go any farther in relating the contents of this book; and only add the wiſh, that the author may find leiſure to continue his plan.

---

*Ed. Jenner Diſquiſito de Cauſis et Effectibus Variolarum Vaccinarum ex Anglico ſermone in Latinum converſa,* ab ALOYS CARENO, M. et Ph. D. 1799, Viennere; with four mezzotinto plates.

Dr. Jenner's firſt publication, "*Inquiry, &c.*" has already been tranſlated into German, by Dr. Ballhorn, of Hanover; but the preſent preſent Latin tranſlation contains alſo his "*further obſervations,* and is faithfully done, though the Latin is not always the beſt. Dr. Odier's, of Geneve, experiments with the vaccine inoculation are added, and likewiſe ſome others, where the inoculation ſucceeded, and ſubſequent infections with ſmall-pox matter did not take. The plates are well executed.

